# Cutaneous application of SecurePig® FLASH, a Pig appeasing pheromone analogue, facilitates adaptation and manages social behavior during feeding in semi-extensive conditions

**DOI:** 10.1186/s40813-024-00363-z

**Published:** 2024-03-05

**Authors:** Manon Chasles, Míriam Marcet-Rius, Jen-Yun Chou, Eva Teruel, Patrick Pageat, Alessandro Cozzi

**Affiliations:** 1 Research Institute in Semiochemistry and Applied Ethology (IRSEA), Quartier Salignan, 84400 Apt, France; 2 Pig Development Department, Animal & Grassland Research and Innovation Centre, Teagasc, Moorepark, Co. Cork P61 C996, Moorepark, Ireland; 3 Institute of Animal Welfare Science, University of Veterinary Medicine Vienna, Veterinärplatz 1, 1210 Vienna, Vienna, Austria

**Keywords:** Agonistic behavior, Appeasing pheromones, Coping, Communal feeding, Pig welfare, Outdoor enclosure, Miniature pigs

## Abstract

**Background:**

Farm animals face several challenges throughout their lives, which can affect both their welfare and their productivity. Promoting adaptation in animals is one way of limiting these consequences. In various animal species, the use of maternal appeasing pheromones is efficient to reduce aggressiveness, improve adaptation and thus ensuring better welfare and productivity. This study sought to investigate the efficiency of a treatment with a Pig Appeasing Pheromone (PAP) on the behavior of pigs reared under semi-extensive conditions and exposed to a potential conflict– collective feeding. Animals (*n* = 14 divided in 2 groups of 7) were subjected to 3 different phases, (A) baseline - no pigs received the PAP, (B) SP − 2 out of the 7 pigs per group received the PAP and (C) AP– all pigs received the PAP. Behaviors related to feeding, aggression and locomotion were compared between the 3 phases of the study.

**Results:**

Compared to the baseline period, we observed that the number of head knocks was reduced when some pigs (*p* < 0.001) and all pigs (*p* < 0.005) received the PAP. Similarly, we observed that the number of fleeing attempts was reduced when some pigs (*p* < 0.001) and all pigs (*p* < 0.001) were treated when compared to baseline. This number was lower in the AP phase than in the SP phase (*p* < 0.001). When all pigs were treated (AP), we also observed that they spent less time investigating the floor than during the two other phases (*p* < 0.001), but they seemed more likely to leave the feeder due to the presence or behavior of another pig of the group (SP vs. AP, *p* < 0.05).

**Conclusions:**

The PAP application improved adaptation in pigs by reducing aggressiveness and promoting conflict avoidance. Those results validate the efficiency of the pheromonal treatment under semi-extensive rearing conditions to help pigs to cope with a challenging situation. Using PAP in the pig industry seems interesting to limit unwanted consequences of farm practices on animal welfare and productivity, by promoting their adaptation.

## Background

Wild and domestic animals are confronted throughout their lives with stress factors of different kinds, such as environmental stress, disease, predation, and social pressure [1]. Animals that, in response to stressors, exhibit inappropriate behaviors see their fitness greatly reduced in comparison to animals that are more flexible in their behavior and show better adaptation skills [2, 3]. The term coping has been defined to characterize those animals that are adapting to a stressor by adopting a behavioral response that enable a diminution of the physiological consequences of this stressor [4, 5].

For farm animals, husbandry practices represent challenges they must face, and we know that some of those practices can have a negative effect on animal health and welfare [6]. Efforts can be made to improve animal welfare by rethinking farming practices to adapt them to animals’ needs. Nevertheless, certain practices, such as abrupt weaning, mixing, or transportation, part of the routine farm management to improve herd performance and efficiency, and will be difficult to completely eradicate. Consequently, even in farming systems that aim to meet the physical and mental needs of animals, such as extensive farming, animals still face challenges, and their welfare will depend on their ability to adapt to these challenges [1] and their capacity to manage conflicts of interest in relation to different resources. These situations can compromise the benefit of group living especially when escalate to aggression [7].

Helping animals cope and adapt better to farming conditions could be an approach to limit stress-related consequences on animal health, behavior, welfare, and productivity. In the swine industry, we know that different practices are related to increased stress and/or aggressive behavior in individuals. On farms, weaning occurs abruptly at around 1 month of age instead of gradual weaning at around 4 months of age under natural and semi-natural conditions [8]. This fact is known to cause significant amount of stress in piglets [9]. Throughout their lives, they will be mixed with unfamiliar pigs, which is a common practice at many stages of production to have homogenous groups and thus facilitate farm management. Nevertheless, this is known to increase fighting and decrease growth [10, 11]. Transportation has been linked to an increase in cortisol in pigs, which is a sign of stress [12]. In addition to those livestock practices, housing type and feeding methods can also induce conflicts in pigs that can increase the level of stress and aggression [13, 14]. Hence, more agonistic behaviors were observed around feeding in pigs fed altogether than in pigs that were fed individually in stalls [13]. All of this is not without consequences for farmers as stress has negative impacts on both the health and welfare of pigs, as well as on their production parameters [15]. Thus, helping pigs to better cope with those farming practices could contribute to modulate their impact on pigs’ behavior. In a feeding context, this could help reduce conflicts between pigs and thus reduce fighting, improve their welfare, and ensure more equal access to food for each individual, supporting better weight gain, thus, its performance.

Thanks to the growing knowledge of animal chemical communication, the use of pheromones, or pheromonotherapy, has been developed to help animals adapt and cope with stress factors. The behavioral and physiological effects of the use of maternal appeasing pheromones have been described in various species. Maternal pheromones are secreted during lactation to attract the young and stimulate suckling in mammals [16], in dogs and cats, they are described as calming [17] and in rabbits, they are known to be learning facilitators [18]. The use of this type of pheromone is known to be efficient in reducing aggressiveness but also in helping animals adopt a better behavioral response when exposed to a stressful situation, as stated in dogs and cats [19–22]. Similarly, in horses, the use of an equine appeasing pheromone can be used to help horses better cope with a stressful situation or a learning task [23, 24]. In farm animals, the use of pheromonotherapy has been reviewed [25], and its use is known to improve animal welfare but also production parameters in rabbits [26] and chickens [27–29]. In both dairy and beef cattle, the strategy to use a cutaneous application of the pheromone enables each individual to carry and spread the appeasing message, leading to improved welfare and production parameters [30–33].

In pigs, a Pig Appeasing Pheromone (PAP) has been identified and is known to be secreted by the sebaceous glands between the mammary chains of the suckling sow. Its behavioral and physiological effects on pigs have been extensively investigated in different situations and in animals of various ages. These various studies highlighted the potential of PAP to help pigs cope with stressful situations, indeed the PAP has been described to reduce agonistic behaviors at mixing in mini-pigs and commercial breeds [34–37], and signs of stress during transport, with a reduction in cortisol secretion [38] and a lower heart rate in treated pigs [39]. Reduction of agonistic behavior when using the PAP has been established in piglets [40], weanling pigs [34, 37], and adult sows [41], underlining the versatility of using pheromonotherapy in the swine industry. Thus, by improving coping abilities, PAP can modulate the negative consequences of modern livestock practices on the physiology and behavior of pigs. However, all studies cited above have been performed on pigs housed indoors and currently we have no data on the effectiveness of this treatment in pigs housed in outdoor, extensive conditions. Moreover, to our knowledge, there is no information on the efficacy of this treatment on the behavior of a socially stable group of adult pigs, in which the hierarchy is well established and where agonistic behaviors are mainly observed during conflicts.

Our study sought to investigate the impact of using a Pig Appeasing Pheromone (PAP), SecurePig® FLASH (SIGNS Labs, France), on the behavior of pigs raised in semi-extensive conditions during a potential conflict, namely the competition for feed access. For this purpose, we subjected stable groups of mini-pigs housed outdoors with ample space a potential conflict of collective feeding, which is known to promote agonistic interactions between animals [13]. Mini-pigs were treated or not with the appeasing pheromone, and their subsequent behavior at feeding was analyzed. Application was cutaneous, enabling each mini-pig to carry and diffuse the pheromonal message. In summary, the different aims of this study were (i) to characterize the possibility of using SecurePig® FLASH (SIGNS Labs, France) on pigs raised outdoors, (ii) to evaluate the product’s efficiency in facilitating pigs’ adaptation to collective feeding, thus reducing agonistic interactions between pigs and (iii) to investigate the efficiency of treating only a few pigs within a group.

## Methods

This study protocol was performed in compliance with the European directive 2010/63/EU on the protection of animals used for scientific purposes and was approved by the French Ministry of Research and the ethical committee for animal experimentation (C2EA125, France, n° 25098-202004091550975).

### Animals

Experimentation was conducted in two consecutive months on 14 adult mini-pigs (*Sus scrofa dosmesticus*), divided in 2 comparable groups of 7. Each group was composed of 3 females and 4 castrated males, and all animals were 7 years-old. These pigs were purchased from Specipig, a mini-pig center for breeding and biomedical research based in Barcelona (Spain) and are the result of a crossbreeding between conventional breeds, Landrace and Large-White, with Asian miniature breeds, Vietnamese and Chinese.

The groups were constituted 2 years before the experiment took place, ensuring that they form socially stable groups and both were housed outdoors, in two identical, adjacent pens of 150 m² providing free access to water, shade, and shelters. Animals were fed twice a day from Monday to Friday and once in the morning on Saturday and Sunday with an appropriate diet for mini-pigs. Animals’ health and welfare were guaranteed by close monitoring by animal keepers and veterinarians thanks to a monthly Animal Welfare Assessment performed by the Animal Welfare Body of the Research Institute [42].

### Experimental design

**Fig. 1:**
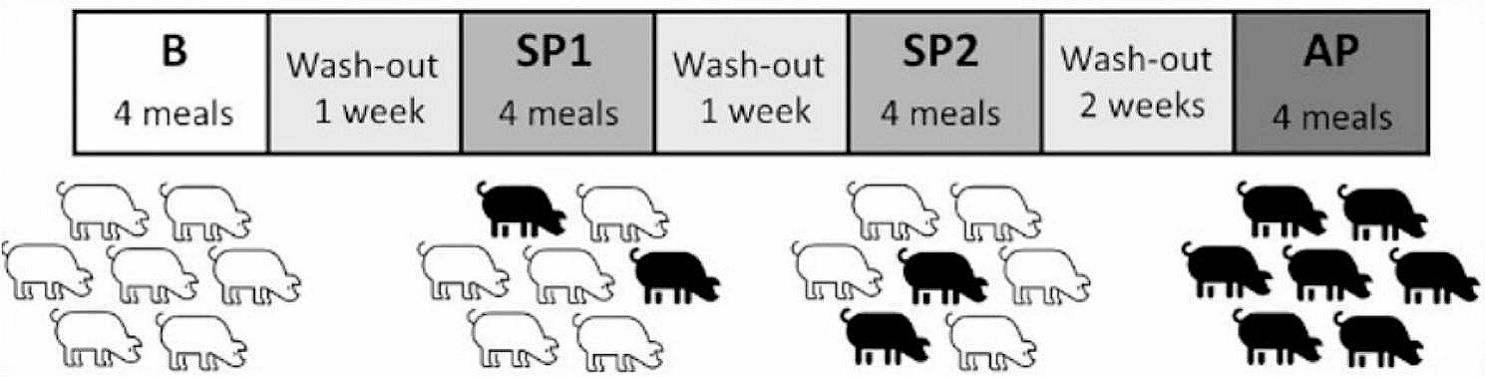
Experimental schedule for one group of 7 pigs

Experimentation was divided in three distinct phases (Fig. 1.): in phase 1, “Baseline” (B), no pigs were treated; in phase 2, called “Some Pigs” (SP), we treated two pigs from each group with the Pig Appeasing Pheromone (PAP; SecurePig® FLASH (SIGNS Labs, France)); in phase 3, called “All Pigs” (AP), all pigs were treated with the same pheromone. During the SP phase, we treated one randomly selected dominant and one subordinate pig (see details in the treatment application section), thus, to limit as much as possible the potential effect of individual behavioral variations in our results, phase 2 (SP) has been performed twice with different pigs treated on replicates SP1 and SP2. This study was conducted in parallel in the two groups of 7 pigs, pigs from each group being submitted to the same procedure at the same time. A wash-out of at least 1 week was scheduled between each experimental phase to ensure that no PAP residuals remained on pigs’ skin.

According to the manufacturer, the product SecurePig® FLASH (SIGNS Labs, France) has a remanence of 5 days, thus, a minimum of 7 days of wash-out was scheduled between the two SP replicates and between the SP phase and the AP phase. This was made to ensure that, at the beginning of a new phase, no residue remained on the pigs previously treated. For the baseline (B) and the AP phase, we analyzed four consecutive meals For the SP phase 8 meals were analyzed for each pen as the SP phase has been performed twice with different pigs treated at each replicate. Each meal was analyzed for 5 min each, for each phase we recorded two morning and two evening feedings, the first feeding analyzed always being an evening one.

Throughout the experiment, the animals were fed solid pellets that were soaked in water for 30 min before feeding to rehydrate them and create a soup that was easier for the mini-pigs to eat. Once the food was ready to be served, the keeper entered the first pen and walked directly to the feeder without interacting with the pigs, when all the pigs were around the feeder the food was distributed and the keeper left calmly. After leaving the first pen, the keeper executed the same procedure in the second pen. The feeder was long enough for all pigs to eat at the same time. Analysis of animals’ behavior began when food started to be distributed and lasted for 5 min.

### Habituation

This experimentation required pigs to be fed collectively but as those pigs were previously fed individually to control food intake and limit aggression between individuals, one month before the experiment began, animal keepers started to feed the pigs collectively. This has been done to ensure all pigs were habituated to this new feeding method and participated in it. During this period, pigs were also habituated to the presence of the cameras and of the investigator behind the fence in front of the feeder to ensure it would not disturb them during data collection.

### Treatment application

The treatment was applied following the manufacturer’s instructions, hence, 5mL of the product was applied on the wither skin of each mini-pig. In this study, we used single-use syringes filled either with PAP (SecurePig® FLASH, SIGNS Labs, France) or a placebo having the same aspect, odor, and texture as the original treatment and which is composed of the vehicle of the SecurePig® FLASH. During the SP phase, the investigator did not know which mini-pig was treated with PAP or placebo. The treatment was applied in the morning at 9 a.m., with the first meal analyzed being the evening meal of the same day.

As the hierarchical position of the pig influences its behavior and interactions with conspecifics [43], we took this information into account, hence for each group of 7, we defined the 3 more dominant and the 3 more subordinate pigs, the remaining pig was considered intermediate. Hierarchy ranking was determined by analyzing 4 feedings prior to the baseline during the habituation period, the level of expression of both aggressive and submissive behaviors as well as the time spent eating were considered. For each of the SP1 and SP2 phases, we randomly selected one dominant and one subordinate pig to be treated simultaneously. The identification of all selected pigs was blinded to the experimenters who applied the PAP and conducted the behavior observation and statistical analysis.

### Behavioral analysis

The behavior of the mini-pigs was analyzed for the first 5 min of each meal, starting when the keeper started to drop the feed in the feeder. A camera was put in front of the feeder and behaviors were then analyzed using the BORIS software [44]. Behaviors observed were divided into 3 categories: Aggression, Feeding, and Locomotion (Table 1). For aggression, both the actor and the receiver of the action were noted, e.g., for the flee behavior, the pig that fled was considered as the actor, and the pig that caused it to flee was considered as the receiver. Behaviors were either analyzed as a frequency (f) or duration (d) as stated in Table 1. Climate conditions, such as temperature and rain were noted for each meal, in order to take this into account if it could impact pigs’ behavior.

**Table 1:** Description of the behaviors observed during the video analysis

Category	Behavior	Description
Aggression*	Bite (f)	The pig bites or attempts to bite another pig
	Flee (f)	The pig quickly avoids another pig (implies running)
	Displace (f)	The pig makes another pig to move by its approach or behavior (mainly bites and head knocks).
	Head knocks (f)	The pig is performing head movement towards another pig with or without contact [45]
**Feeding**	Eat (d)	The pig has the head in the feeder
	Stop Eating by itself (f)	The pig stop eating without the involvement of any perturbation (ref)
	Stop Eating by another pig (f)	The pig stop eating due to another pig’s active or passive intervention (ref)
	Floor Investigation (d)	The pig makes movements with its nose on the floor
**Locomotion**	Walk (d)	The pig is moving in any direction

*identity of the actor and receiver are noted; (f) for frequency; (d) for duration

### Statistical analysis

Data analysis was realized thanks to R version 4.2.2 [46] and RStudio version 1.4.1103 and statistical significance was set at *p* < 0.05.

### Main analysis: comparison between the three phases (baseline, SP and AP)

For all analyses, we used mixed models for repeated measures, with the initial model including the phase (baseline, SP as the mean of SP1 and SP2, AP) as a fixed effect and the pig, the group, the number of the feeding (1 to 4), and the climate conditions as a random effect. This model was then simplified as best as possible regarding AIC and BIC criteria.

For behaviors “Eat”, “Walk”, and “Floor investigation”, they were expressed as durations, hence we planned to perform a General Linear Mixed Models (GLMM) analysis. The assumptions of normality and homoscedasticity of model residues were assessed. For the normality, the Q-Q plot and the histogram were plotted, and the Kolmogorov-Smirnov tests was carried out. For the homoscedasticity, the scatterplot of the predicted values and the standardized residuals was displayed, and the Levene test was produced. As assumptions were verified, GLMM were performed on raw data. For behaviors “Bite”, “Flee”, Displace”, “Head knock”, “Stop eating by itself”, and “stop eating by another pig”, they were expressed as frequencies, hence we planned to perform Generalized Linear Mixed Models (GzLMM) with the Poisson distribution. The dispersion of the data has been evaluated by looking at the Pearson X²/DF indicator, validating the feasibility of this analysis.

In both cases, if a significant effect of the phase was observed, multiple comparisons were performed using the Tukey adjustment method.

### Supplementary analysis: principal component analysis (PCA) for the phases baseline and AP

To detect the possible effects of the treatment with PAP on pigs’ behavioral profiles, we performed a principal component analysis (PCA). According to results previously obtained on the comparison of the 3 phases: baseline, SP, and AP; we decided to perform the PCA only for the phases “baseline” and “AP”. As one condition to perform PCA is the sample independence, each phase has been analyzed separately and within each one we analyzed the mean of each behavior over the four meals. Firstly, we included all variables and individuals in the PCA analysis of both phases, but many variables were antagonistic, as for example “bite actor” and “bite receiving”, thus we were not able to isolate any profile other than dominant or subordinate pig. Hence, we decided to analyze dominant and subordinate pigs and their respective behaviors separately. Neutral pigs were represented in both analyses. For each variable, the contribution to the construction of the axis, the correlation, and the cos² were assessed. Variables with a poor quality of representation (cos²<0.5) were interpreted with caution.

## Results

### Effect of the PAP treatment on pigs’ behavior: comparison of baseline with SP and AP phases

To assess the effect of the appeasing pheromone on pigs’ behavior, we compared the results obtained during the baseline (no pig treated) with the results from the phases SP and AP, when some or all the pigs were treated.

**Fig. 2:**
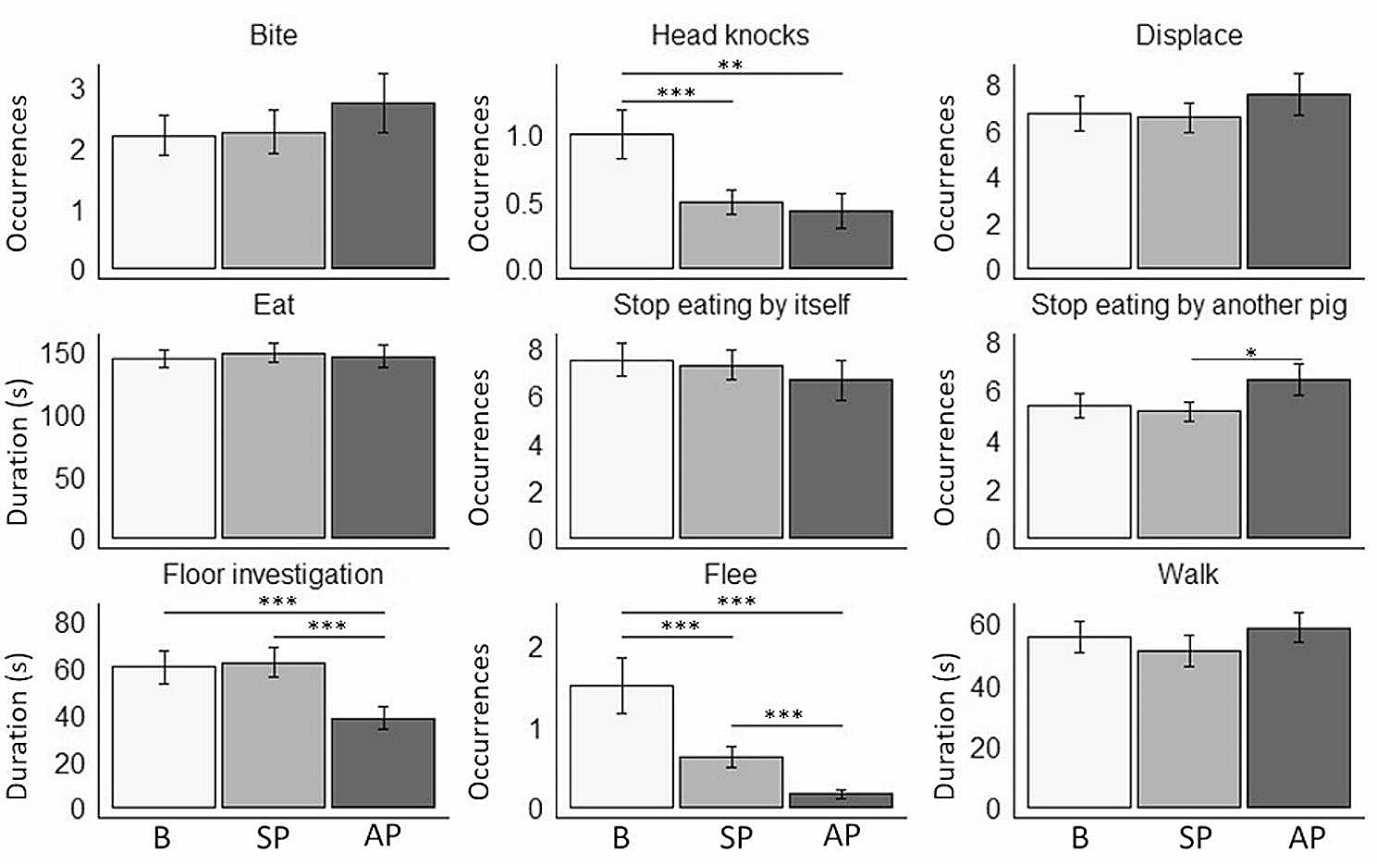
Mean pig behavior for 4 consecutive meals on each of the 3 treatment phases (B, SP, AP). Animal behavior was compared before treatment (B, baseline), when some pigs (SP) in each group were treated with PAP or when all pigs (AP) in each group were treated with PAP. Data are presented as mean ± standard deviation. Stars indicate significant differences between phases according toto the Tuckey based multiple comparisons. (*p < 0.05, **p < 0.01, ***p < 0.001). *N* = 14 pigs for each phase

Regarding agonistic interactions **(**Fig. 2.**)**, we found that pigs exhibited more head knocks during baseline than during SP (GzLMM, Tukey’s Post hoc test, *p* < 0.001) and AP phases (GzLMM, Tukey’s Post hoc test, *p* < 0.01). We have also observed a decrease in the number of fleeing attempts in pigs treated with PAP (SP and AP phases) in comparison with our observations during the baseline phase (GzLMM, Tukey’s Post hoc test, *p* < 0.0001). No effect of the treatment has been found on the behaviors “bite” (GzLMM, X²=4.00, DF = 2, *p* = 0.13) and “displace” (GzLMM, X²=4.28, DF = 2, *p* = 0.12).

Concerning feeding dynamics **(**Fig. 2.**)**, no effect on the time spent eating has been observed (GLMM, X²=0.12, DF = 2, *p* = 0.94), and pigs did not stop eating due to another pig during the AP phase (GzLMM, Tukey’s Post hoc test, *p* = 0.06). Floor investigation behavior was indeed impacted by the PAP treatment **(**Fig. 2.**)**, as pigs spent less time exploring the ground during AP treatment than during baseline (GLMM, Tukey’s Post hoc test, *p* < 0.001). No effect of the treatment has been found on the behaviors “stop eating by itself” (GzLMM, X²=4.05, DF = 2, *p* = 0.13) and “walk” (GLMM,X²=5.33, DF = 2, *p* = 0.07).

### Effect of number of pigs treated: comparison between SP and AP phases

To detect if the number of pigs within a group that are treated with PAP has an impact on the behavior of the whole group, we compared the results obtained during the SP phase, when only 4 pigs out of the 14 were treated, with the results of the AP phase, when all pigs were treated simultaneously.

Regarding agonistic interactions **(**Fig. 2.**)**, we found that PAP treatment reduces the number of head knocks, while no difference was detected between SP and AP phases (GzLMM, Tukey’s Post hoc test, *p* = 0.99). Nevertheless, we observed a decrease in the number of fleeing attempts when all pigs (AP) were treated compared to when only some pigs (SP) were treated (GzLMM, Tukey’s Post hoc test, *p* < 0.001).

Concerning feeding dynamics **(**Fig. 2.**)**, our experimentation showed that when all pigs (AP) were treated they were more likely to stop eating due to another pig than the SP phase (GzLMM, Tukey’s Post hoc test, *p* = 0.05). The number of pigs per group that were treated with the appeasing pheromone, also impacted the behavior “floor investigation”, as when all pigs (AP) received the treatment, they spent significantly less time exploring the ground than when only some pigs (SP) were treated with the PAP (GLMM, Tukey’s Post hoc test, *p* < 0.0001).

### Analysis of behavioral profiles by PCA

**Fig. 3:**
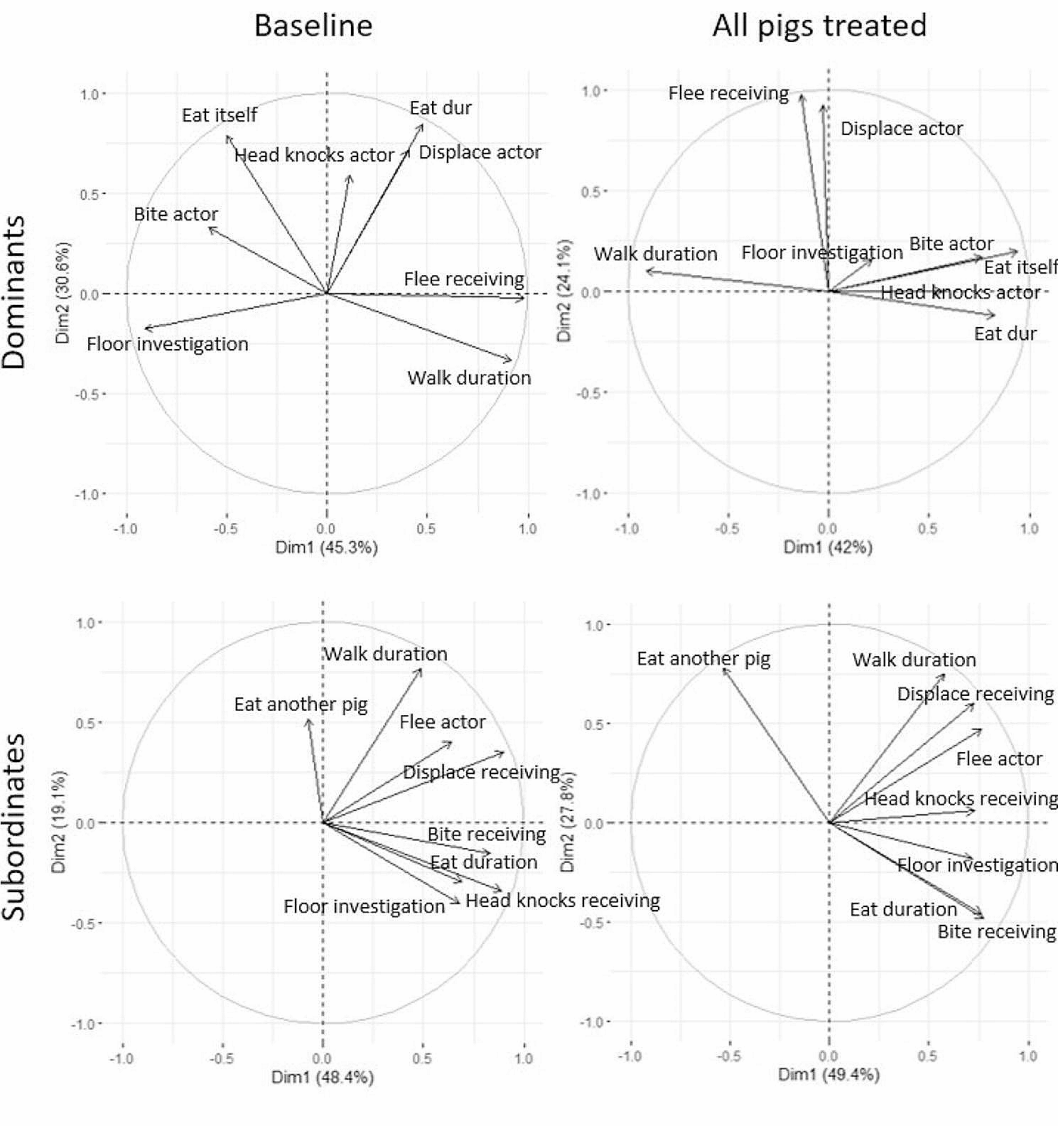
Correlation circles for the two principal components for dominant and subordinate pigs, with and without PAP treatment. PCA was performed separately for dominant and subordinate pigs, neutral pigs were considered in both analyses. PCA was only done in two phases, baseline, when no pigs were treated, and AP, when all pigs were treated. Arrows represent the correlation between each variable and the axis. In each PCA, the behavior of 8 pigs was analyzed

Four PCA were performed to analyze separately dominant and subordinate pigs as their behavior were antagonistic but also to analyze separately the two phases “baseline” and “AP” as PCA analysis require sample independence. The correlation, contribution, and cos² of each variable for the two principal components and for each of our four PCA analyses are shown in Table 2 and are graphically represented in Fig. 3.

**Table 2:** Parameters for the first two principal components (PC1 and PC2) for the Baseline and AP phases

	Baseline								All Pigs Treated (AP)						
**Dominants**	PC1 (45.3%)				PC2 (30.6%)				PC1 (42%)				PC2 (24.1%)		
	Corr.	Cont.	Cos²		Corr.	Cont.	Cos²		Corr.	Cont.	Cos²		Corr.	Cont.	Cos²
**Bite_actor**	**-0.589**	**9.57**	**0.347**		0.327	4.37	0.107		**0.764**	**17.36**	**0.583**		0.168	1.47	0.028
**Head-Knocks_actor**	0.112	0.34	0.013		**0.589**	**14.19**	**0.347**		**0.565**	**9.49**	**0.319**		0.001	0.00	0.000
**Displace actor**	0.407	4.56	0.165		**0.716**	**20.94**	**0.512**		-0.029	0.02	0.001		**0.924**	**44.31**	**0.853**
**Flee receiving**	**0.979**	**26.42**	**0.958**		-0.023	0.02	0.001		-0.135	0.55	0.018		**0.977**	**49.59**	**0.955**
**Walk dur**	**0.918**	**23.24**	**0.843**		-0.337	4.65	0.114		**-0.910**	**24.64**	**0.828**		0.101	0.52	0.010
**Eat dur**	0.475	6.21	0.225		**0.846**	**29.26**	**0.716**		**0.824**	**20.21**	**0.679**		-0.120	0.74	0.014
**Eat itself**	-0.503	6.96	0.253		**0.786**	**25.27**	**0.618**		**0.941**	**26.39**	**0.886**		0.197	2.02	0.010
**Floor investigation**	**-0.907**	**22.68**	**0.823**		-0.178	1.30	0.032		0.212	1.34	0.045		0.161	1.35	0.026
**Subordinates**	PC1 (48.4%)				PC2 (19.1%)				PC1 (49.4%)				PC2 (27.8%)		
	Corr.	Cont.	Cos²		Corr.	Cont.	Cos²		Corr.	Cont.	Cos²		Corr.	Cont.	Cos²
**Bite receiving**	**0.831**	**17.81**	**0.690**		-0.155	1.58	0.024		**0.772**	**15.10**	**0.596**		-0.481	10.41	0.232
**Head kocks receiving**	**0.889**	**20.40**	**0.790**		-0.339	7.51	0.115		**0.727**	**13.39**	**0.529**		0.060	0.16	0.004
**Displace receiving**	**0.899**	**20.87**	**0.809**		0.353	8.15	0.124		**0.726**	**13.34**	**0.527**		0.599	16.11	0.358
**Flee actor**	0.637	10.48	0.406		0.402	10.57	0.162		**0.762**	**14.71**	**0.581**		0.472	9.99	0.222
**Walk dur**	0.487	6.12	0.237		**0.766**	**38.37**	**0.586**		0.575	8.37	0.331		**0.749**	**25.19**	**0.561**
**Eat dur**	**0.691**	**12.34**	**0.536**		-0.295	5.70	0.087		**0.764**	**14.79**	**0.584**		-0.458	9.42	0.210
**Eat another pig**	0.074	0.14	0.006		0.516	17.44	0.266		0.535	7.25	0.286		**0.779**	**27.24**	**0.606**
**Floor investigation**	0.677	11.83	0.459		-0.404	10.67	0.163		**0.718**	**13.05**	**0.515**		-0.181	1.47	0.033

In bold, the most relevant traits for each principal component (PC).

Corr.: the correlation score to each component.

Cont.: the contribution of each variable to the component (in %).

Cos²: the quality of the representation.

### Dominant pigs– baseline

Seven components were extracted from the analysis, only the two first were interpreted as they explained 75.9% of the total variation (Table 2). The first component (PC1) explained 45.3% of the behavioral variation and was mainly correlated with behaviors involving a locomotion activity (walk, flee receiving, floor investigation). The second component accounted for 30.6% of the behavioral variation and was mainly correlated with meal-related behaviors (displace actor, eat, stop eating by itself).

### Dominant pigs– AP

Seven components were extracted from the analysis, only the two first were interpreted as they explained 66.1% of the total variation (Table 2). The first component (PC1) explained 42% of the behavioral variation and was mainly correlated with meal-related behaviors (eat, stop eating by itself), aggressive behavior (bite actor), and locomotor activity (walk). The second component accounted for 24.1% of the behavioral variation and was mainly correlated with behaviors related to the provocation of a movement by the focal pig on another pig (displace actor, flee receiving).

### Subordinate pigs– baseline

Seven components were extracted from the analysis, only the two first were interpreted as they explained 67.5% of the total variation (Table 2). The first component (PC1) explained 48.4% of the behavioral variation and was mainly correlated with aggression and avoidance (bite receiving, flee actor, head-knock receiving, displace receiving) and food-related behavior (eat, floor investigation). The second component accounted for 19.1% of the behavioral variation and was mainly correlated with walking behavior.

### Subordinate pigs– AP

Seven components were extracted from the analysis, only the two first were interpreted as they explained 77.2% of the total variation (Table 2). The first component (PC1) explained 49.4% of the behavioral variation and was mainly correlated with aggression and avoidance (bite receiving, flee actor, head-knock receiving, displace receiving) and food-related behavior (eat, floor investigation). The second component accounted for 27.8% of the behavioral variation and was mainly correlated with two behaviors “walk” and “stop eating by another pig”.

## Discussion

This study had different goals, investigating the effects of Pig Appeasing Pheromone (SecurePig® FLASH, SIGNS Labs, France) in a potential conflict among a socially stable group of pigs, testing the efficiency of the pheromonal treatment in semi-extensive conditions, and determining if treating only some pigs within a group could be enough to have a significant impact on pigs’ behavior.

Our study highlighted that when treated with PAP, pigs’ behavior at feeding was modified. We observed a decrease in the number of head knocks performed by the pigs but also in the number of fleeing attempts. Those results are consistent with previous studies showing that a treatment with PAP reduced the occurrence of agonistic interactions in different contexts in adults and young, in mini-pigs and commercial pigs [34–37]. This suggests that pigs subjected to a conflict, adopt a calmer behavior when treated with the appeasing pheromone than when untreated. For the flee behavior, it was interesting to note that the more pigs were treated, the less the behavior was expressed. During our experimentation, we did not find any effect of the PAP on the duration of feeding which seems is in contradiction with the results obtained previously showing that PAP stimulates feeding behavior and ensures a better weight gain in treated piglets when compared to control ones [40]. Nevertheless, we had to mention that these results were obtained in pigs of less than one month, at weaning, and under conventional indoor housing, hence under experimental conditions highly different to ours, as we were working on adults raised under semi-extensive conditions. On the other hand, the dynamics of the collective feeding have indeed been modified. In our study, we observed that when all pigs were treated (AP phase), they were more likely to stop eating in the feeder due to the presence or behavior of another pig than when only some pigs received the pheromonal treatment. We observed a trend for this behavior, even if not significant, to be more expressed when all pigs were treated than when no pigs were treated. Interestingly, we observed that, when all pigs were under a PAP treatment, they were more likely to stop their eating behavior consequently to another pig approach. But at the same time, we observed a decrease in the number of agonistic interactions and no increase in the total number of pigs displaced. Hence, we cannot explain this increased propensity to leave the feeder by an increase in the aggressiveness of the surrounding pigs, but we could suggest that when exposed to the pheromone, pigs were more careful about other pigs’ behavior. As a previous study demonstrated that pigs were able to anticipate a potential threat [49], it is possible that the application of PAP improved this ability. Hence, we suggest that pigs treated with PAP were better at managing interactions and anticipating conflicts and so were able to eat in a calmer way. This is highly interesting as from previous studies, we know that the way pigs eat, i.e., calm or stressed, can impact their performance by reducing their weight gain [47, 48]. Those results were confirmed by the PCA analysis which highlighted that before the PAP treatment, behaviors “displace” and “head knock” of dominant pigs were correlated while it is no longer the case when all pigs were treated. This hypothesis is consistent with a previous study showing that, in the context of encounters with unfamiliar mini-pigs, pigs treated with PAP were less aggressive and tended to spend more time looking at each other [36]. As in our experiment we were simultaneously analyzing the behavior of 7 pigs, it was not possible for us to determine whether two pigs were having a reciprocal look.

Concerning the floor investigation behavior, a type of exploratory behavior, we observed that it decreased when all pigs received the PAP treatment compared to when no pigs or only some pigs were treated with the pheromone. Our hypothesis to explain this result is that the floor investigation behavior could be in our case a redirection of the eating behavior; indeed pigs were only exploring the floor around the feeder, possibly looking for food. We believe that pigs were performing this behavior to access food without having to put their head in the feeder where most of the agonistic interactions took place. For pigs that have been displaced from the feeder, looking for food out of the feeder could be seen as a safer option than directly going back to the feeder. When all pigs were treated with the appeasing pheromone, they better understood the intentions of other pigs and were therefore calmer. As a result, they were less likely to avoid the feeder and search for food on the ground around it. As stated before, pigs can anticipate a threat [49]. In our situation, under the PAP treatment, pigs were maybe better at anticipating a putative conflict with another pig. This could have helped them be calmer and maybe less anxious around the feeder. By consequences they were less likely to avoid the feeder and to look for food on the ground.

Hence, our results not only give interesting information on the positive impact of a PAP treatment on the behavior of pigs at collective feeding, but also gives crucial data on the method of application to ensure the efficiency of the pheromonal intervention. Indeed, we observed several behavioral differences when all pigs were treated compared to the results obtained after treating 2 out of the 7 pigs per group. Pigs showed fewer fleeing behaviors and spent less time exploring the floor when they were all treated than when only a part of the group was treated. Conversely, they were more likely to stop eating due to another pig’s presence. As stated above, we suggest that these behavioral changes are the sign of a better assessment by the pig of the behavior and intentions of the surrounding pigs and a calmer reaction to it. According to our results, it seems that treating all pigs in a group is imperative to have the best results on animal behavior and to promote a better adaptation of the whole group to a conflict, which may in turn avoid the possible impact of stress on the weight gain during fattening phases [48]. But we cannot rule out the possibility that we had better results treating all pigs because in the SP phase we were not treating the right pigs or just not enough pigs. We made the choice to treat at the same time one dominant and one subordinate pig out of the 7 pigs of the group. Since the mechanism of action of the maternal pheromone in reducing agonistic behavior is not clearly established, maybe treating all dominants or all subordinates could have led to different conclusions. Although we know that the PAP has an appeasing role, what is unknown is if it reduces aggressiveness in a treated pig or if it reduces the potential for a treated pig to be the target of aggression. Indeed, the PAP treatment is applied on the wither skin of pigs, and it lasts around 5 days. Given these parameters, the maternal pheromone may influence the behavior of the treated pig, but also on the behavior of the surrounding pigs which may have access to the pheromone by interacting with the treated pig. If the pheromone acts directly on the treated pig, decreasing its aggressiveness, treating all dominant pigs could have been more efficient. Conversely, we can also hypothesize that the PAP while applied on the skin of a subordinate pig improves its social skills and at the same time sends a message to the surrounding pigs not to assault it. In this case, treating only the subordinate pigs could be a possibility. But it is also likely that the PAP acts both ways and that we had significantly fewer results in the SP phase because we only treated less than 30% of the pigs in each group, which was not enough. Since the pheromone is applied on the skin, each pig treated becomes a transmitter of the appeasing message to its congeners. In our context of semi-extensive rearing conditions, the density of pigs is low which make it more difficult for each pig to receive the pheromonal message if only some pigs have been treated. To clarify these hypotheses, further studies are needed, in which we might treat different quantities of pigs per group or treat pigs according to their hierarchical status, all dominants versus all subordinates for example. Having more information about the action mechanism of the PAP could help us provide a good practice guide to farmers who want to apply it to improve animal welfare on farms.

## Conclusion

Results of the present study suggest that a cutaneous application of PAP is efficient in semi-extensive conditions to induce beneficial changes in the behavior of pigs when subjected to a conflict, even in a socially stable group of pigs. It has been efficient in helping pigs to adapt to collective feeding by significantly reducing aggressiveness, promoting conflict avoidance, and thus ensuring calmer pigs. Whereas the effect of the commercial form of the PAP, SecurePig® FLASH (SIGNS Labs, France) was previously validated in improving animal welfare and reducing aggressiveness, we validated for the first time its efficiency on semi-extensive conditions. Although further studies are needed to confirm this, based on current results, treatment of all pigs in a group seems mandatory to achieve maximum effect on animal behavior and guarantee calmer pigs during feeding.

We believe that this could be an interesting tool for the pig industry to help animals better adapt to challenges they will face either in indoor or outdoor, intensive or extensive rearing conditions. Better management of conflicts such as competition at feeding could have a direct impact on welfare and performance in the pig industry.

## Data Availability

No datasets were generated or analysed during the current study.

## Abbreviations

AP: All Pigs– phase when all pigs received the treatment
GLMM: General Linear Mixed Models
PAP: Pig Appeasing Pheromone
PCA: Principal Components Analysis
SP: Some Pigs - phase when 2 out of the 7 pigs per group received the treatment
